# Supplementary material for: Recognition of an Ala-rich C-degron by the E3 ligase Pirh2
Source: Nat Commun. 2023 Apr 29;14:2474. doi: 10.1038/s41467-023-38173-6 (PMC10148881; doi:10.1038/s41467-023-38173-6)
Supplement: Supplementary file 1 — Supplementary Information [file 41467_2023_38173_MOESM1_ESM.pdf]

Supplementary information for

Recognition of an Ala-rich C-degron by the E3 ligase Pirh2

Xiaolu Wang[1,2,#], Yao Li[1,3,#], Xiaojie Yan[1,3,#], Qing Yang[3], Bing Zhang[3], Ying Zhang[4], Xinxin Yuan[3], Chenhao Jiang[4], Dongxing Chen[5], Quanyan Liu[6], Tong Liu[7], Wenyi Mi[1,4], Ying Yu[1,2,*] and Cheng Dong[1,3,6,7,*]

*Corresponding author. E-mail: dongcheng@tmu.edu.cn or yuying@tmu.edu.cn

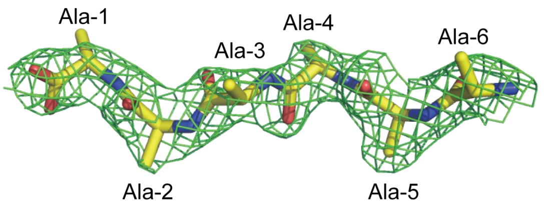

Supplementary Fig. 1 | Fo-Fc omit map of Ala6/C-degron. Electron density is contoured at 1.5 σ level.

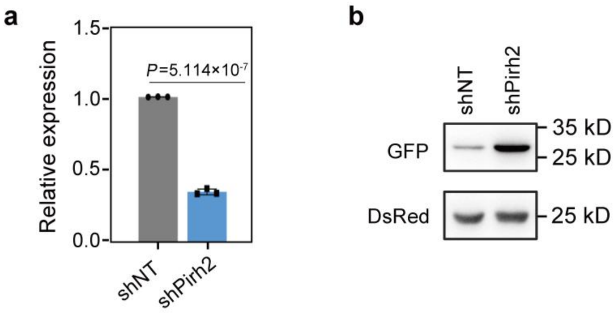

Supplementary Fig. 2 | Knockdown of Pirh2 by shRNA. a The relative mRNA levels of Pirh2 in shNT and shPirh2 cells. The shRNA targets the 3'-untranslated region (UTR) of endogenous *Pirh2*. Error bars indicate S.E.M. of three biological replicates. *P* values were determined using unpaired two-tailed Student's t-tests; n=3 biologically independent samples. **b** Western blot analysis of the change in DsRed and GFP expression levels in Pirh2 knockdown cells, using DsRed as an internal control. Representative images, n=3. Source data are provided as a Source Data file.

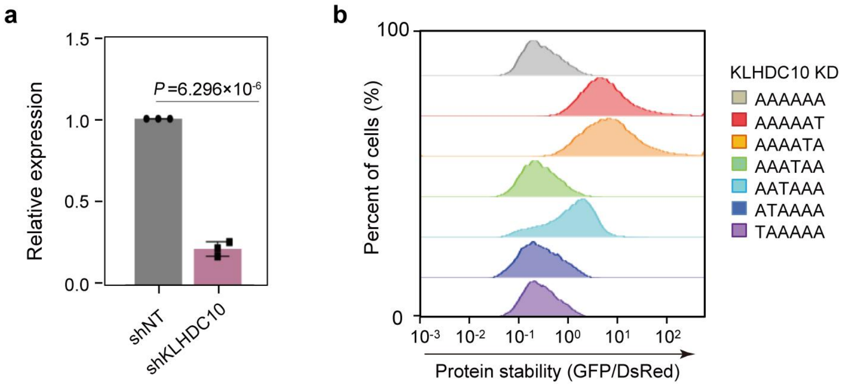

Supplementary Fig. 3 | Stability analysis of different substitutions of Ala6/C-degron in KLHDC10 knockdown cells. a The relative mRNA levels of KLHDC10 in shNT and shKLHDC10 cells. Error bars indicate S.E.M. of three biological replicates. *P* values were determined using unpaired two-tailed Student's t-tests; n=3 biologically independent samples. Source data are provided as a Source Data file. **b** The stability assay of GFP-fused the indicated sequences in HEK293T cells with KLHDC10 knocked down. All FACS sequential gating images are provided in Supplementary Fig. 9.

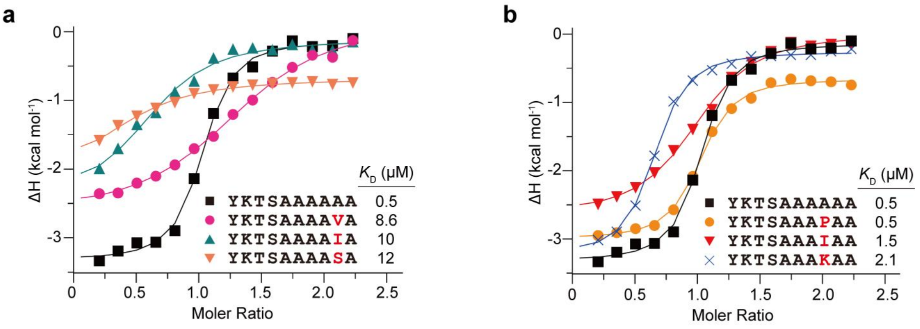

Supplementary Fig. 4 | ITC measurements of binding affinities of Pirh2 to the peptide with position substitutions. a ITC fitting curves of Pirh2 titrated by the peptide with substitutions at position -2. **b** ITC fitting curves of Pirh2 titrated by the peptide with substitutions at position -3. The corresponding peptide sequences and binding affinities (K_D) are indicated.

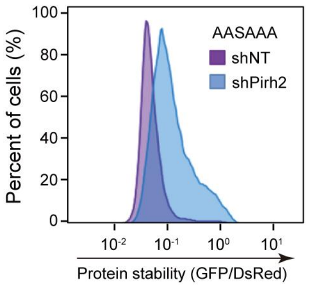

Supplementary Fig. 5 | Stability analysis of GFP-fused A-A-S-A-A-A degron in HEK293T cells with or without shPirh2 treatment. All FACS sequential gating images are provided in Supplementary Fig. 9.

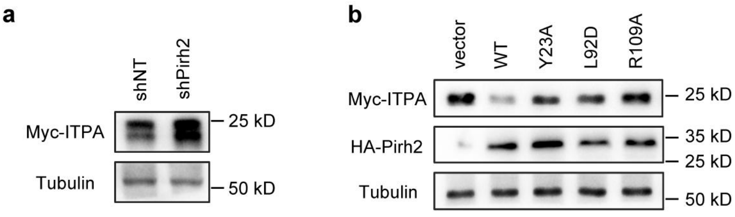

Supplementary Fig. 6 | Stability analysis of full-length ITPA (inosine triphosphate pyrophosphatase) by western blotting. a Stability analysis of Myc-tagged full-length ITPA upon Pirh2 knockdown in HEK293T cells by western blotting. **b** Stability analysis of Myc-tagged full-length ITPA with overexpressed HA-tagged Pirh2 (WT or mutant) proteins in HEK293T cells by western blotting. Representative images, n=3. Source data are provided as a Source Data file.

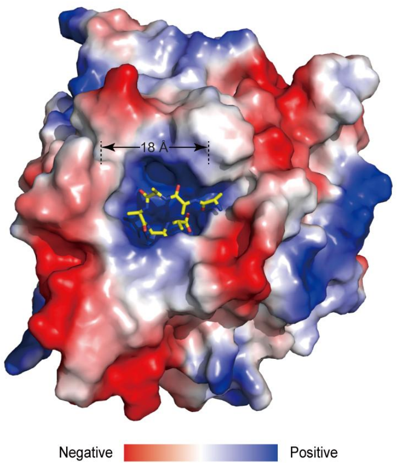

Supplementary Fig. 7 | The electrostatic potential surface of the Ala6/C-degron binding pocket in KLHDC10 (red, negative; blue, positive). The six-Ala peptide was docked into the Alphafold-predicted KLHDC10 structure using the Schrodinger software suite.

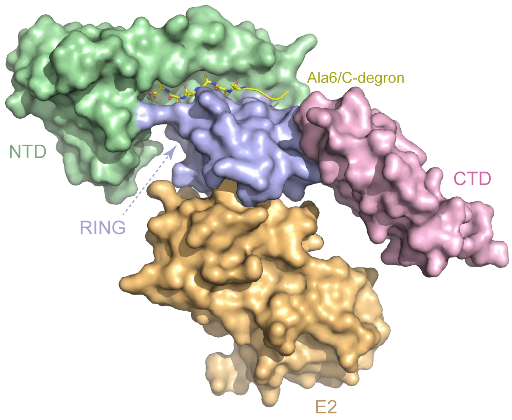

Supplementary Fig. 8 | Binding mode of a potential ternary complex between Pirh2, polyAla/C-degron and ubiquitin conjugating enzyme E2 (UBE2D2). The structure of RNF12 RING domain in complex with UBE2D2 (PDB: 6W9D) is used for the superposition of the Pirh2 RING. PolyAla/C-degron is colored with yellow.

For Fig.4c
AAAAAA

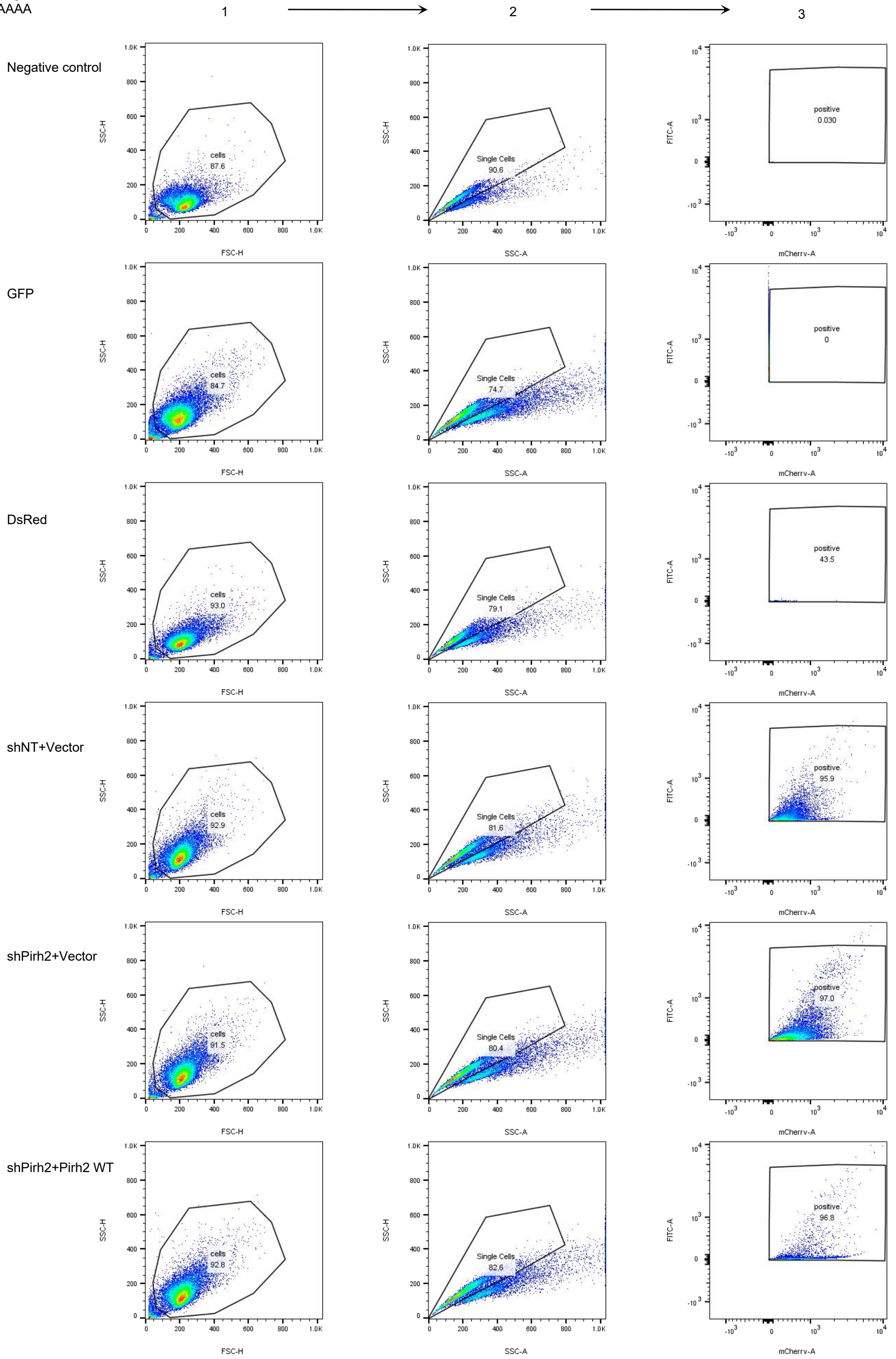

For Fig.4c
AAAAAA

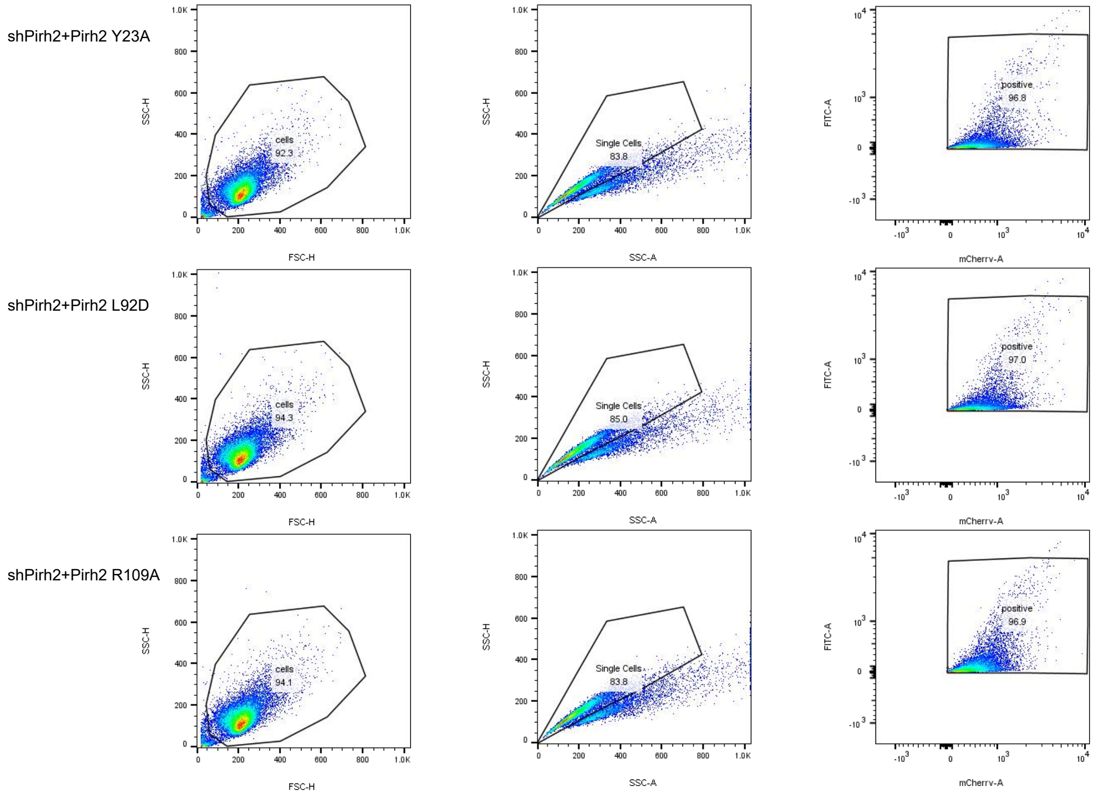

For Fig.6a

Negative control

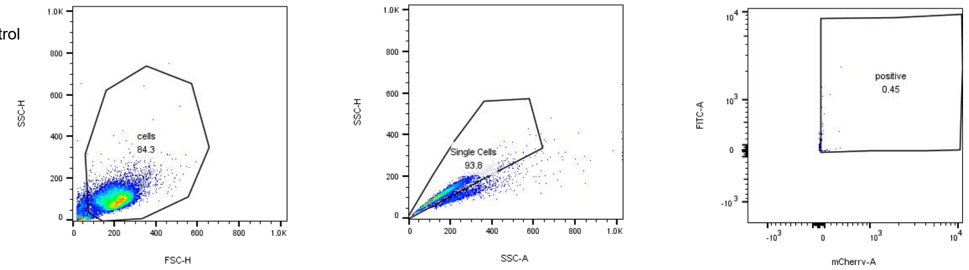

GFP

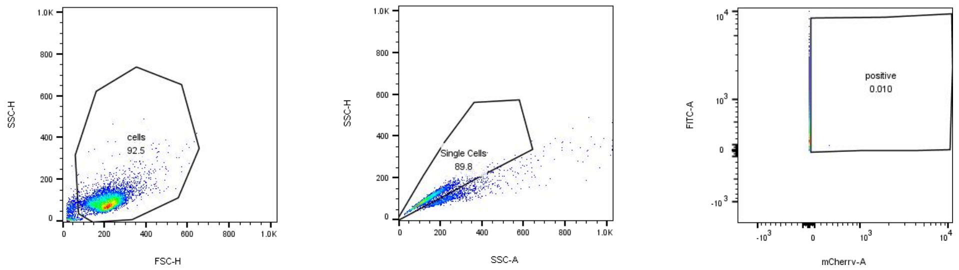

DsRed

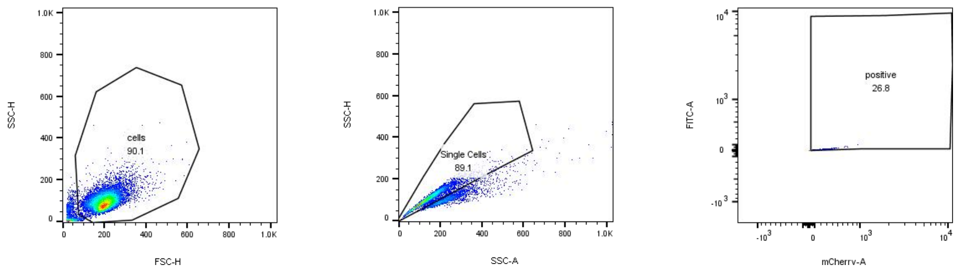

AAAAAA
shNT

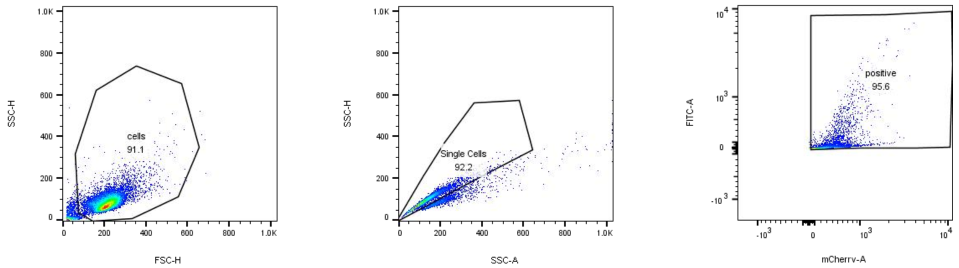

For Fig.6a

AAAAVA
shNT

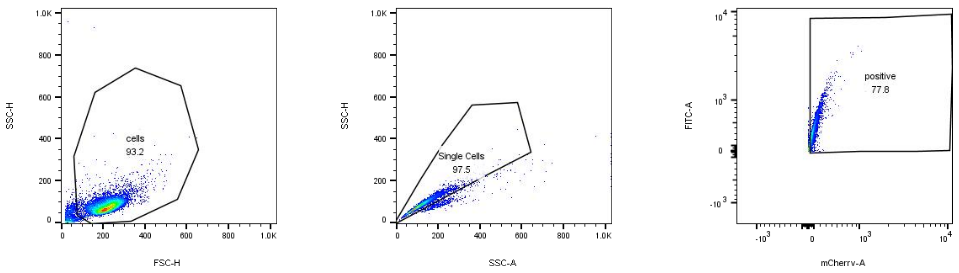

AAAAIA
shNT

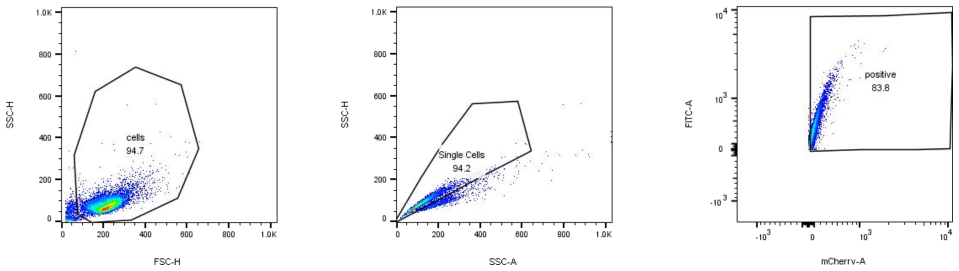

AAAASA
shNT

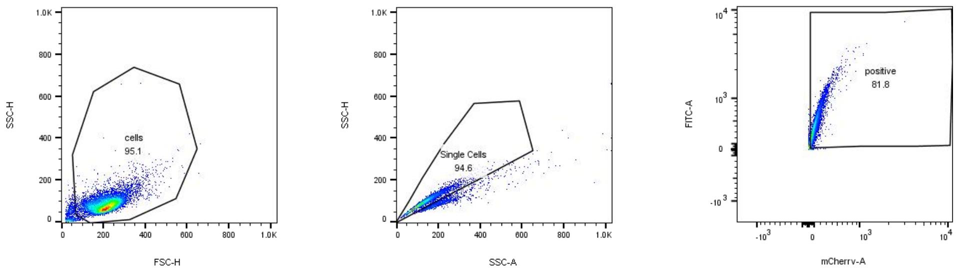

For Fig.6b

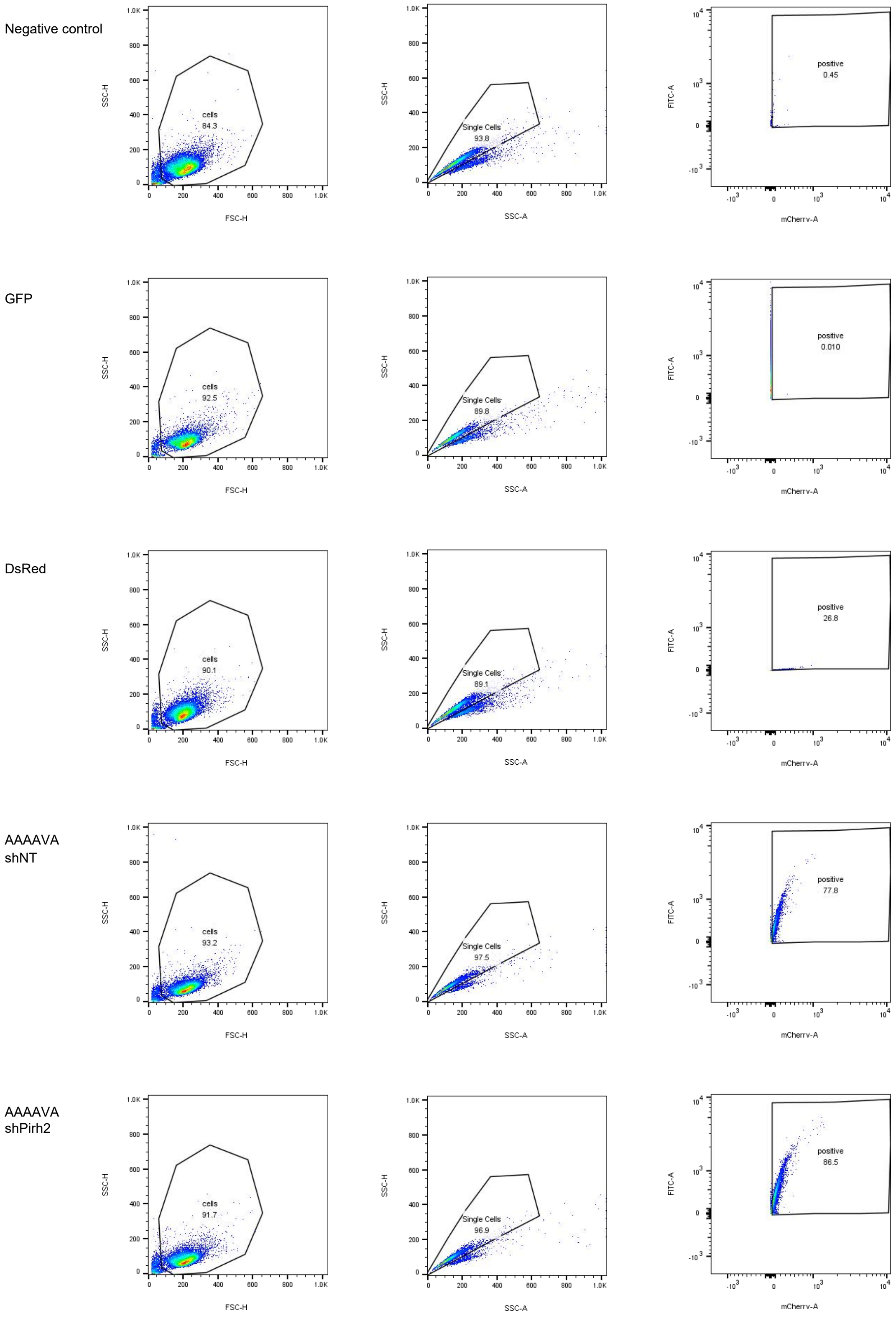
Negative control
cells
84.3
Single Cells
93.8
positive
0.45
GFP
cells
92.5
Single Cells
89.8
positive
0.010
DsRed
cells
90.1
Single Cells
89.1
positive
26.8
AAAAVA
shNT
cells
93.2
Single Cells
97.5
positive
77.8
AAAAVA
shPirh2
cells
91.7
Single Cells
96.9
positive
86.5
FSC-H
SSC-H
SSC-A
mCherrv-A
FITC-A

For Fig.6b

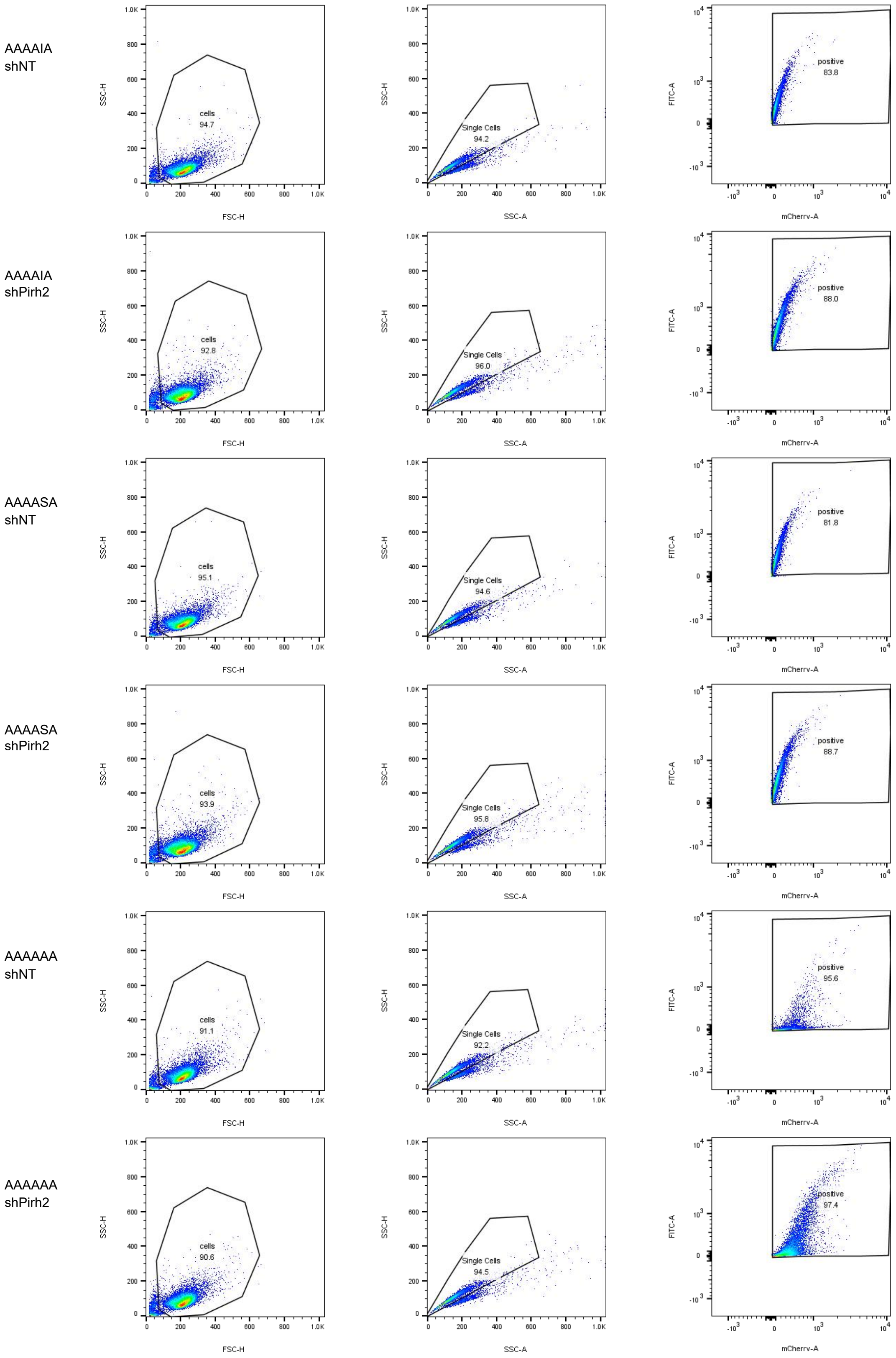
AAAAIA shNT
cells 94.7
Single Cells 94.2
positive 83.8
AAAAIA shPirh2
cells 92.8
Single Cells 96.0
positive 88.0
AAAASA shNT
cells 95.1
Single Cells 94.6
positive 81.8
AAAASA shPirh2
cells 93.9
Single Cells 95.8
positive 88.7
AAAAAA shNT
cells 91.1
Single Cells 92.2
positive 95.6
AAAAAA shPirh2
cells 90.6
Single Cells 94.5
positive 97.4
SSC-H
FSC-H
SSC-A
FITC-A
mCherry-A

For Fig.6c

AAAAAA
shNT

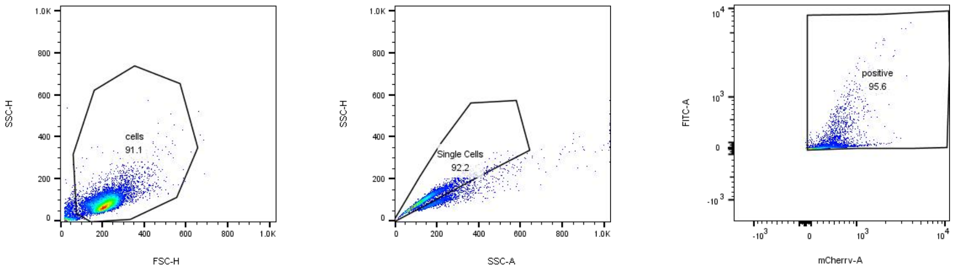

AAAPAA
shNT

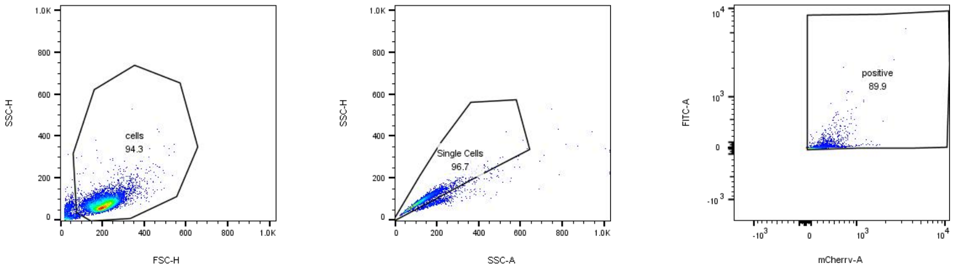

AAAIAA
shNT

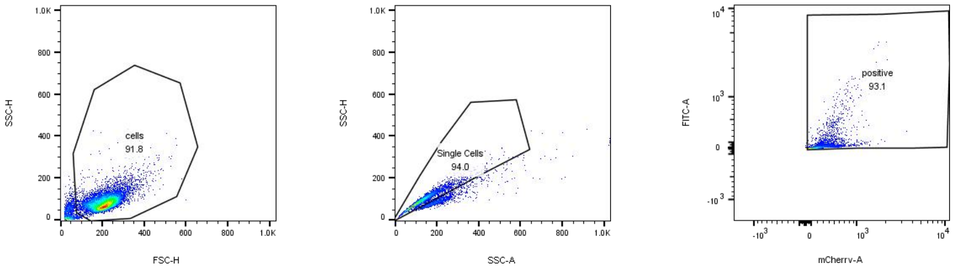

AAAKAA
shNT

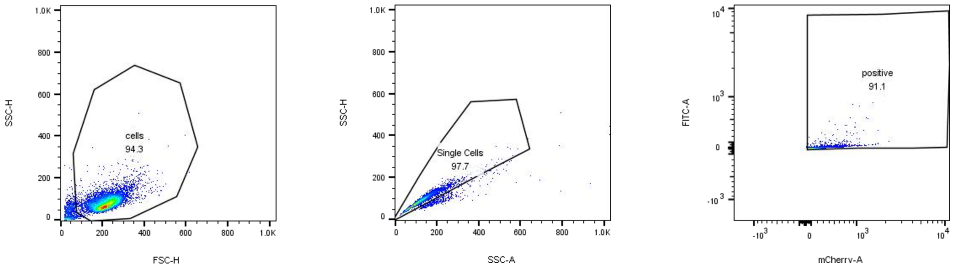

For Fig.6d

AAAPAA
shNT

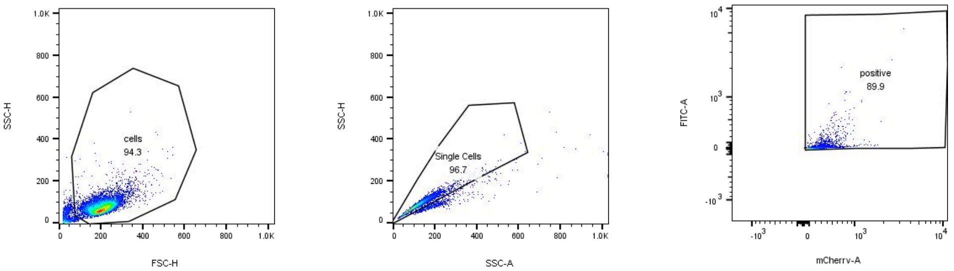

AAAPAA
shPirh2

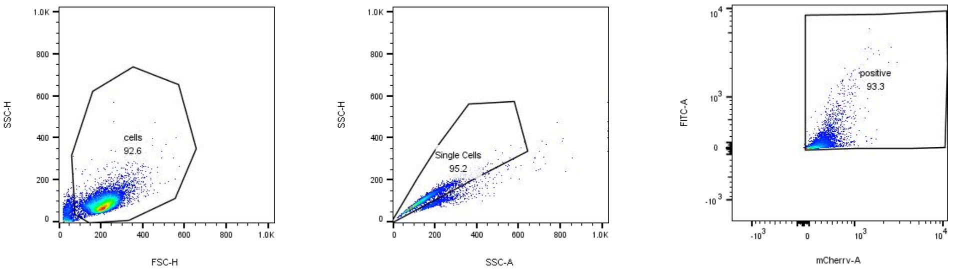

AAAIAA
shNT

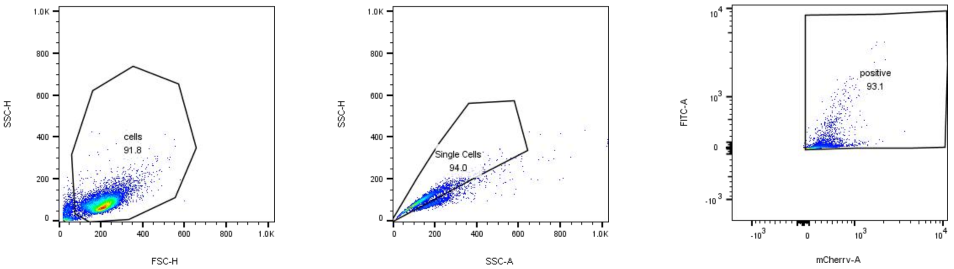

AAAIAA
shPirh2

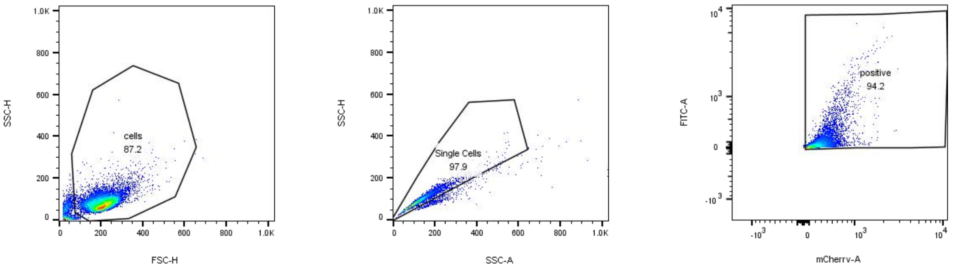

AAAKAA
shNT

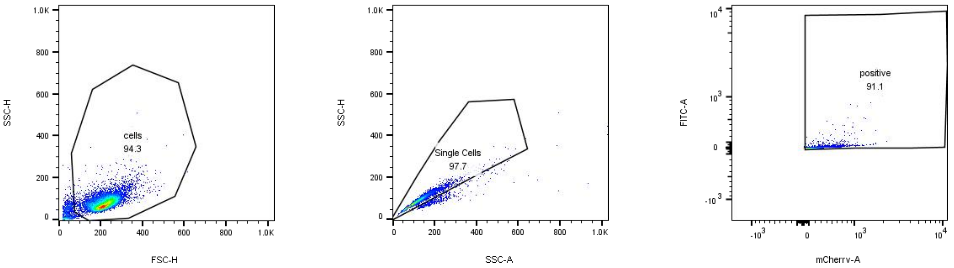

AAAKAA
shPirh2

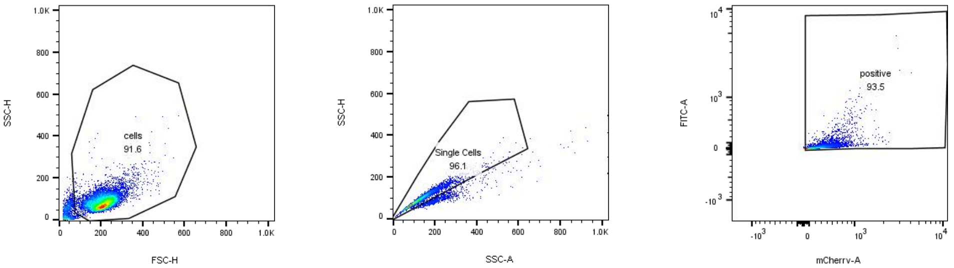

For Fig.6e
AAAIAA

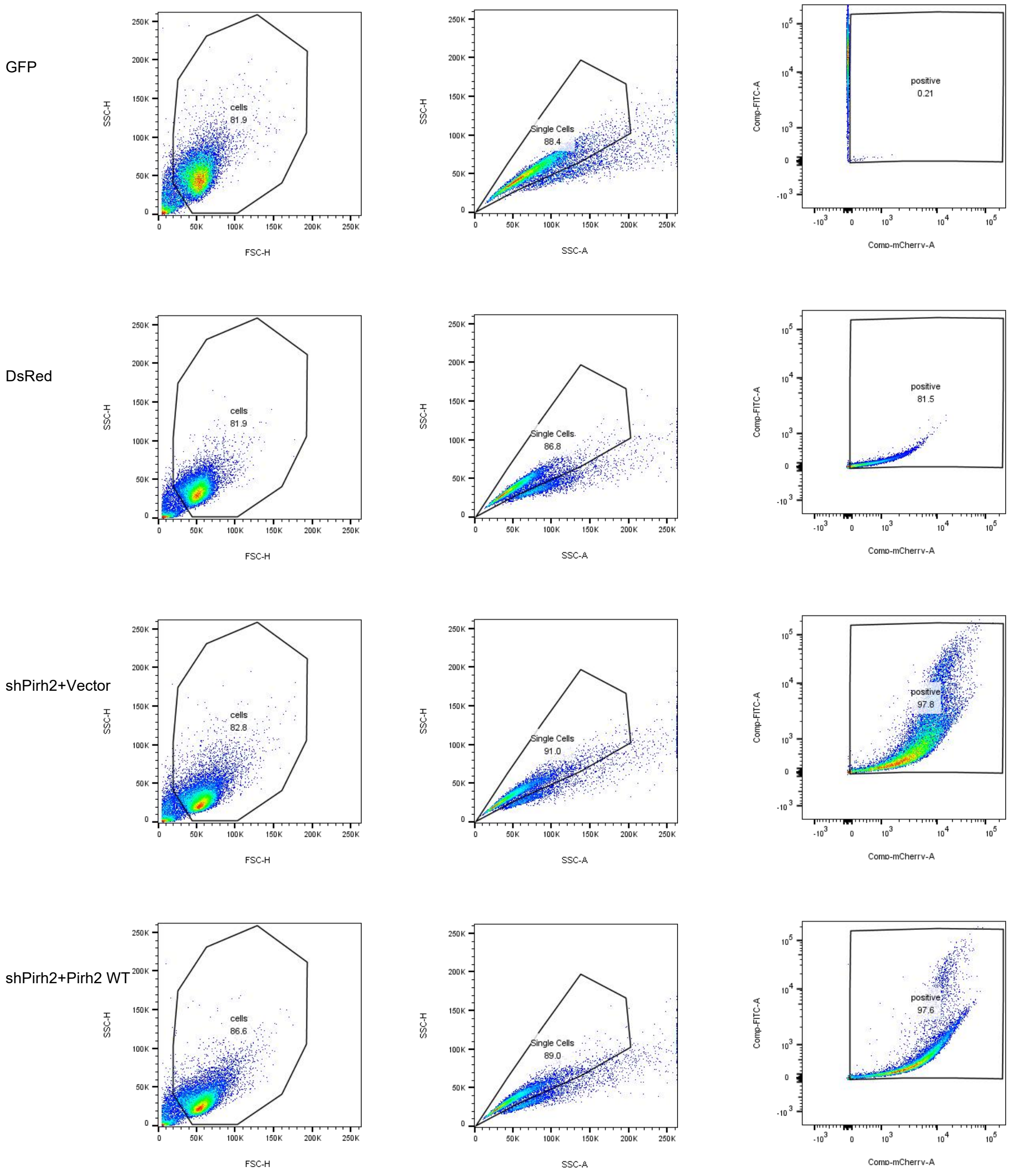

For Fig.6e
AAAIAA

shPirh2+Pirh2 Y23A

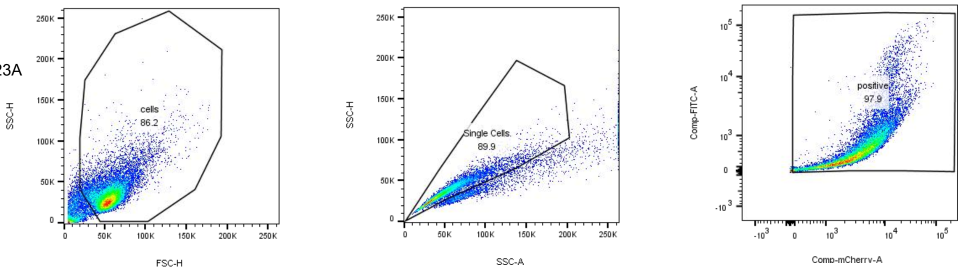

shPirh2+Pirh2 L92D

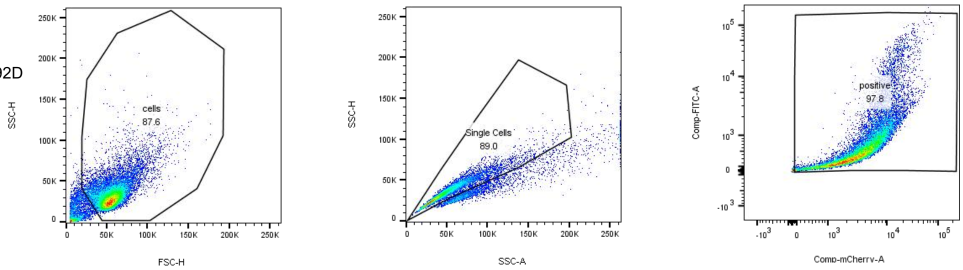

shPirh2+Pirh2 R109A

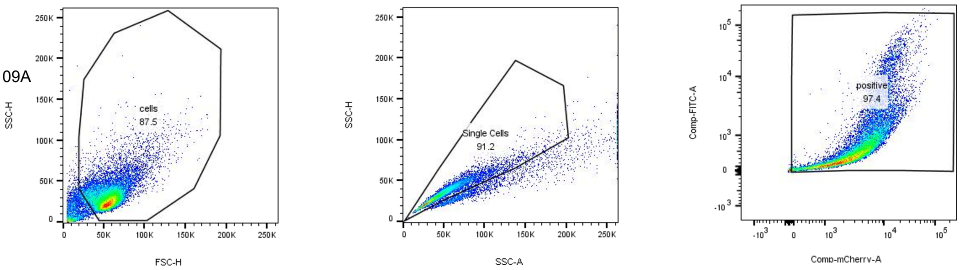

For Fig.6g
AASAAA

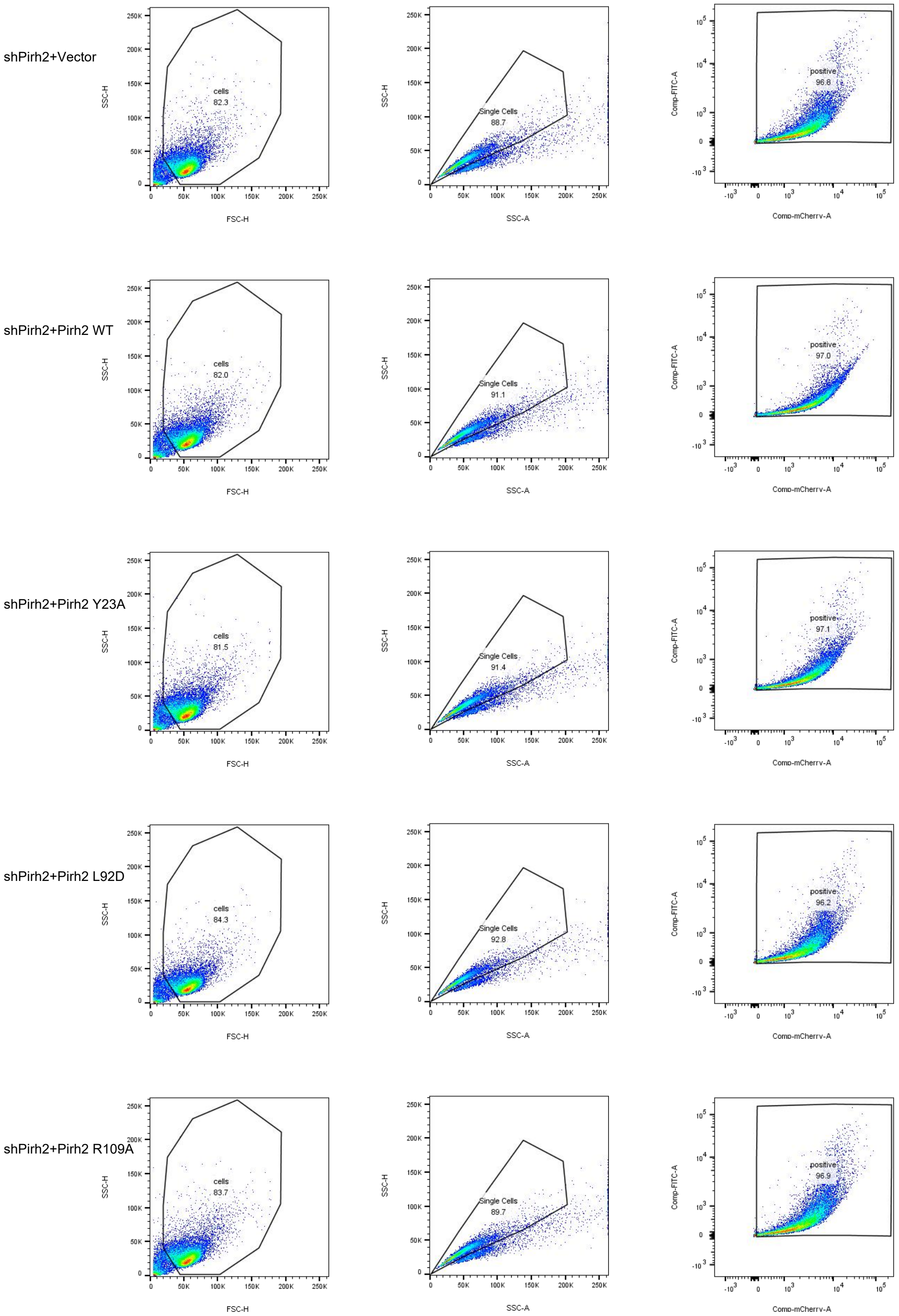

For Supplementary Fig. 3

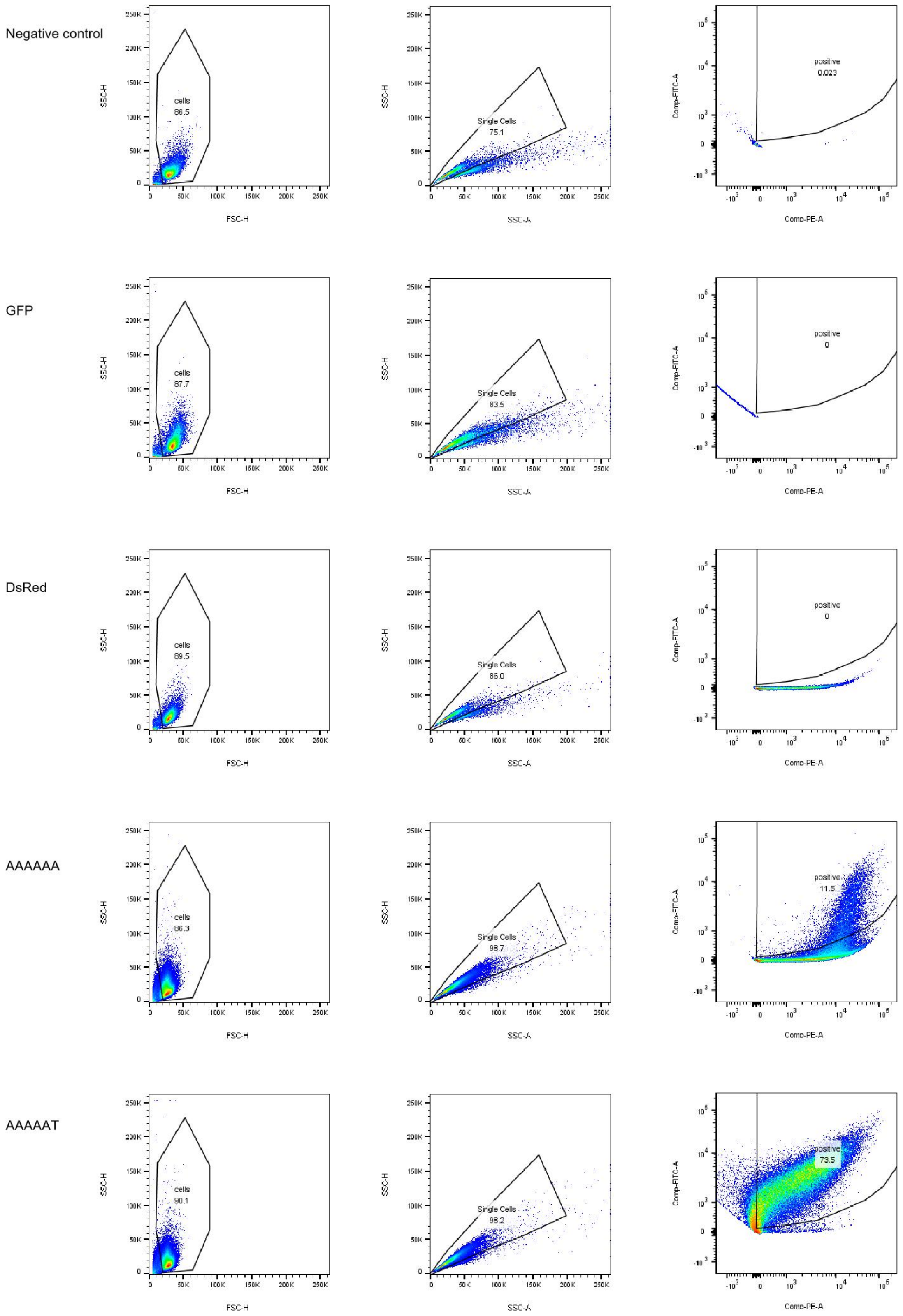

For Supplementary Fig. 3

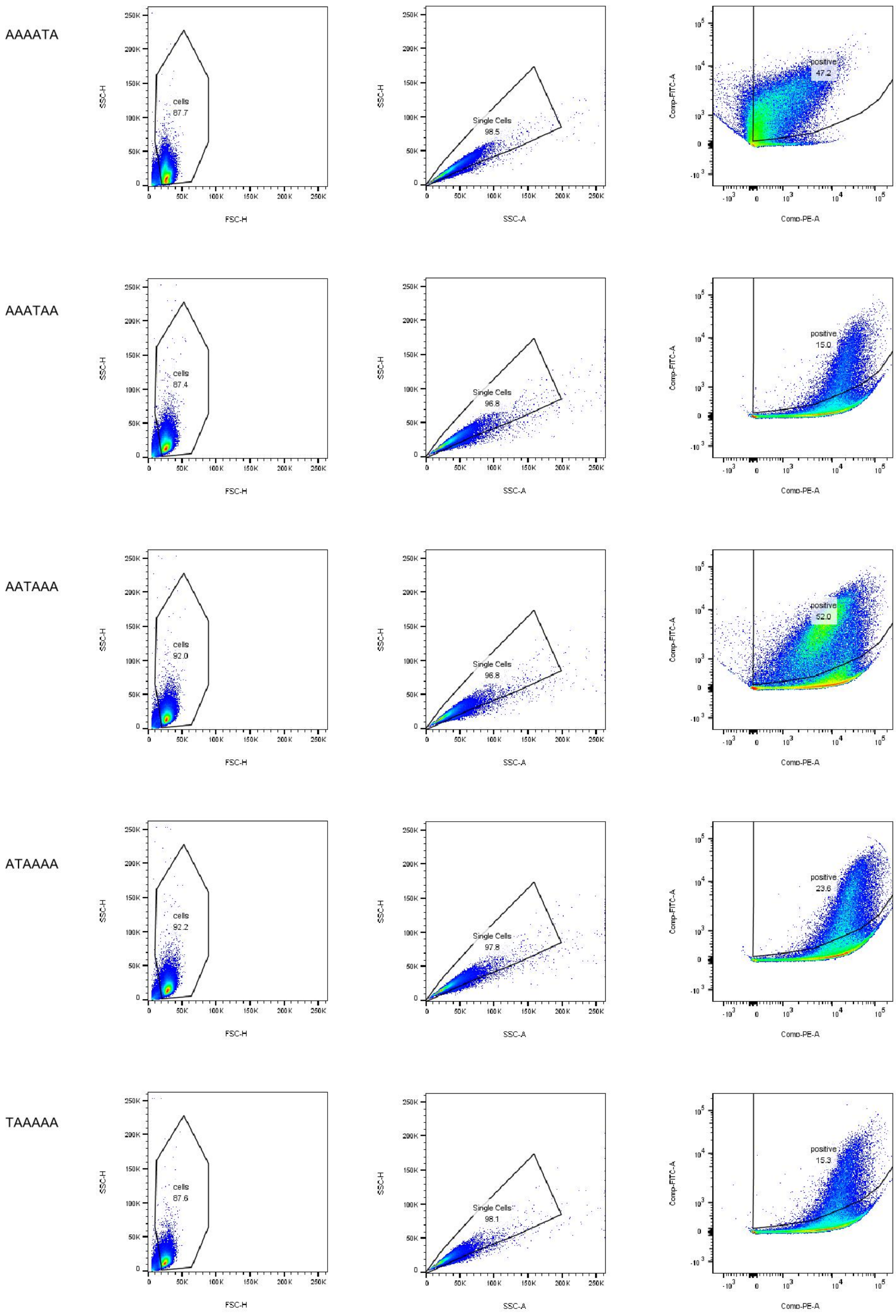

For Supplementary Fig. 5
AASAAA

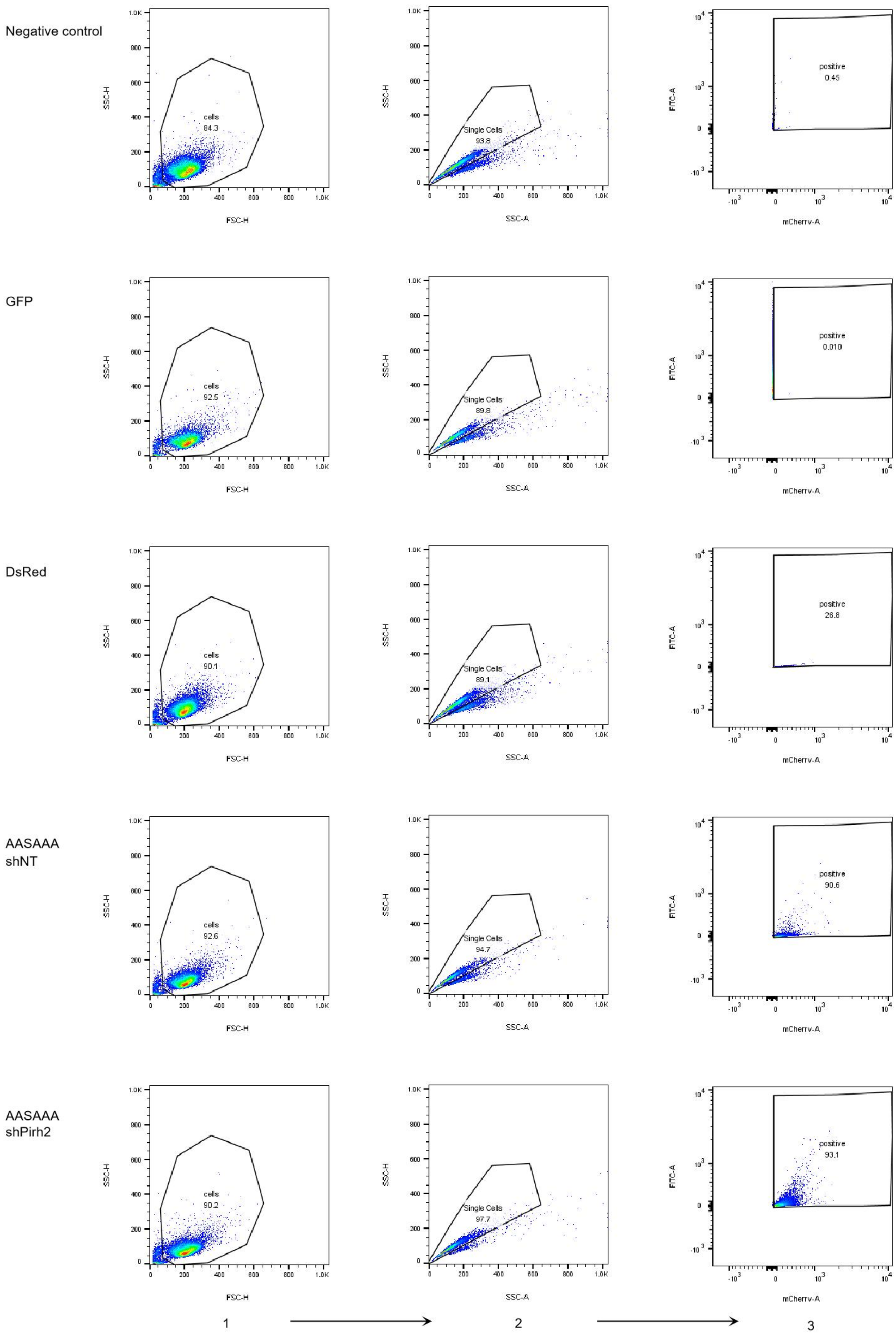

Supplementary Fig. 9 | Gating images for FACS. 1. Gate on FSC-H vs. SSC-H was set to include all cell populations, but excluding debris; 2. Gate on SSC-A vs. SSC-H was set to exclude doublets; 3. The selected cells was calculated for GFP/DsRed ratio.
